# High systemic immune‐inflammation index is relevant to osteoporosis among middle‐aged and older people: A cross‐sectional study

**DOI:** 10.1002/iid3.992

**Published:** 2023-08-29

**Authors:** Suli Zhang, Wenyan Ni

**Affiliations:** ^1^ Department of Operating Room Wujin Hospital Affiliated to Jiangsu University (Wujin People's Hospital) Changzhou Jiangsu China; ^2^ Department of Nursing Wujin Hospital Affiliated to Jiangsu University (Wujin People's Hospital) Changzhou Jiangsu China; ^3^ Wujin Clinical College of Xuzhou Medical University Changzhou Jiangsu China

**Keywords:** middle‐aged and older people, NHANES, osteoporosis, systemic immune‐inflammation index

## Abstract

**Background:**

As one of novel inflammatory indexes proposed in recent years, systemic immune‐inflammation index (SII) can comprehensively reflect the inflammatory and immune state of the body. This study aims to explore the relationship between SII and osteoporosis among middle‐aged and older people.

**Materials and Methods:**

Our study includes 20,497 individuals from National Health and Nutrition Examination Survey (NHANES) 2005–2008, and target study population are confined to people aged 45 years and above. SII is calculated as platelet count × neutrophil count/lymphocyte count. Multivariate logistic regression analysis is used to explore the link between SII and osteoporosis, and receiver operating characteristics curve is used to screen optimal cut‐off value of SII for indicating the occurrence of osteoporosis.

**Results:**

A total of 435 people with osteoporosis are screened among 4625 middle‐aged and older people, and individuals in osteoporosis group have higher SII than those in nonosteoporosis group (*p* = .024). Logistic regression analysis indicates that with the enhancement of SII, prevalence of osteoporosis in each tertile category also increases (*p* < .001). This tendency is also not changed in univariate model (*p* < .001), as well as the adjustments for different parameters. Moreover, we also identify that SII of 530.09 is the optimal cut‐off value for indicating the occurrence of osteoporosis among middle‐aged and older people.

**Conclusions:**

This present NHANES‐based study noticed that higher SII is positively linked to osteoporosis among middle‐aged and older people, and SII should not exceed 530.09 for them to obtain a potentially lower risk of osteoporosis.

## Graphical abstract

This NHANES‐based research indicates that the higher systemic immune‐inflammation index is positively correlated with the risk of osteoporosis in the middle‐aged and older people, and the SII should not exceed 530.09 for them to obtain a potentially lower risk of osteoporosis.

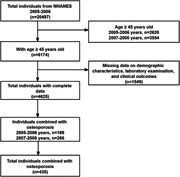

## 1 INTRODUCTION

Osteoporosis is a kind of systemic disease characterized with decline of bone density and bone mass, and the destruction of bone microstructure,^1^ which tends to result in the osteoporotic fractures and is one of the main causes of disability and death in the middle‐aged and older individuals.^2^, ^3^ Meanwhile, osteoporosis and associated secondary hazards also bring heavy economic pressure and burden to patients’ families and social medical service system.^4^ According to a previous report by Ling et al.,^5^ the risk of osteoporotic fractures in the patients with osteoporosis was as high as 40%, while the occurrence of osteoporosis was not easy to be noticed. Of note, most patients are diagnosed after the occurrence of brittle fractures, which delays the most proper time for the prevention and treatment.^6^, ^7^ Therefore, the early screening and diagnosis of osteoporosis is particularly significant.^8^

Currently, the World Health Organization recommends using dual energy X‐ray absorptiometry (DXA) to determine the bone mineral density (BMD) of the test population, and further defines different degrees of BMD as three levels, including the osteoporosis, osteopenia, and normal bone mass.^9^, ^10^ Although the DXA is universally recognized as the gold standard for detecting BMD, which has the advantages of high accuracy and excellent repeatability, while this method is relatively expensive and not suitable for the large‐scale screening.^11^, ^12^ In addition, with the in‐depth researches of osteoporosis, the scholars have indicated that the osteoporosis also belongs to the category of chronic inflammatory diseases. Although the relevant pathophysiological mechanism has not been entirely clarified, the academic community of bone immunity is convinced that the immune system and inflammatory factors play a critical role in the occurrence and development of osteoporosis.^13^ Hence, from the perspective of bone immunity and inflammation, it is promising for us to explore an objective, simple, economic, sensitive, specific, and noninvasive predictor for the early discovery and diagnosis of osteoporosis.

As one of the novel inflammatory indexes proposed in recent years, the systemic immune‐inflammation index (SII) can comprehensively reflect the inflammatory and immune state of the body.^14^ In a previous study by Lu et al.,^15^ SII was defined as a new immune and inflammation evaluation index, which was obtained by the platelet count × neutrophil count/lymphocyte count, and a higher SII indicated that patients had stronger inflammation and weaker immune response. In a previous study, Fang et al.^16^ revealed that the postmenopausal women with higher levels of SII also had a significantly higher risk of postmenopausal osteoporosis. Therefore, given that immune and inflammatory factors may have impact on bone metabolism, it can be theoretically explained that SII has a certain diagnostic value for the osteoporosis. Specifically, the higher levels of neutrophils and platelets, and lower levels of lymphocytes in patients may indicate that they have more inflammatory factors to promote the bone resorption and less protective inflammatory factors to inhibit the bone resorption, which suggests a potentially high risk of osteoporosis.^17^, ^18^, ^19^ Based on this, this current study is aimed to assess the association between SII and osteoporosis in middle‐aged and older people in National Health and Nutrition Examination Survey (NHANES) 2005–2008, thereby providing the reference for the future relevant studies.

## 2 MATERIALS AND METHODS

### 2.1 Source of samples and data

The related samples and data are all obtained from NHANES, which is an ongoing cross‐sectional survey managed by National Center for Health Statistics (NCHS) and designed to evaluate the health condition of noninstitutionalized civilians among United States.^20^ After family interviews, the physical examination is conducted on participants at the mobile examination centers, and the relevant data (including medical status, sociodemographic structure, behavioral condition, and so on) is then recorded and evaluated. The related biological samples (such as blood and urine) of the participants are gathered by the bridle‐wise staffs for next laboratory examination. Moreover, as a simply exempt study, this current study only involves the existing progression data from the NHANES, and is approved by the NCHS Research Ethics Review Board, and the informed consent is also obtained from all participants.

### 2.2 Study design and included population

**Figure 1:**
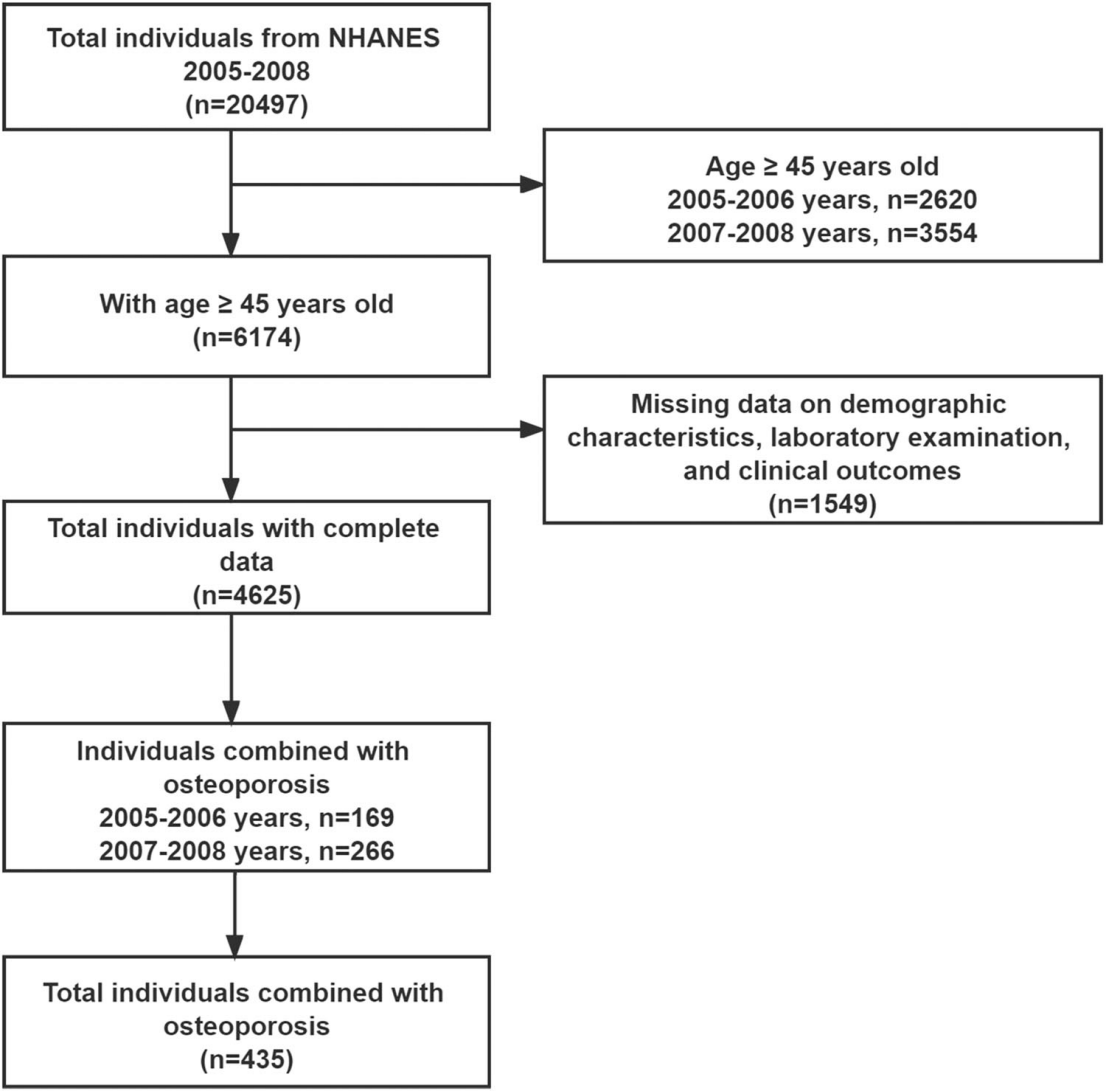
The flow chart of the sample screening in NHANES 2005–2008. NHANES, National Health and Nutrition Examination Survey.

As shown in Figure 1, this cross‐sectional study contains a total of 20,497 individuals from NHANES 2005–2008, and the target study population are focused on the people aged 45 years or older. In this regard, osteoporosis is common in middle‐aged and older people, and is more common in postmenopausal women. According to the international standard division, the middle‐aged people mainly refer to the group between 45 and 59 years old, and the elderly people mainly refer to the group over 60 years old.^21^, ^22^ Based on this, we identify the population included in this study as the age group of 45 years or older. Subsequently, 1549 individuals with the missing data on terms of demographic characteristics, laboratory examination, and clinical outcomes are excluded, and 4625 individuals with intact data are enrolled in next analysis. Parameters that could closely exhibit the medical status, sociodemographic structure, behavioral condition, and laboratory examination of the included individuals are further searched and recorded. Therein, the primary osteoporosis is focused in this study, and any factors related to secondary osteoporosis are excluded (including use of osteoporosis and corticosteroid drugs, hyperparathyroidism, kidney disease, and so on). Furthermore, the included people are divided into the osteoporosis group (*n* = 435) and nonosteoporosis group (*n* = 4190).

### 2.3 Determination of measurement methods and disease definitions

In terms of the measurement of SII, the lymphocyte, neutrophil, and platelet counts are analyzed using the automated hematology analyzing devices and exhibited as ×10^3^ cells/µL, and SII is calculated as platelet count × neutrophil count/lymphocyte count. As for the definition of osteoporosis, the BMD of lumbar spine and femur are measured using DXA (Siemens), and the condition of osteoporosis is evaluated according to the diagnosis from the international diagnostic guidelines,^23^, ^24^ and the diagnostic standard is described as *t*‐score less than −2.5 standard deviation (*SD*). In addition, the definition of combined with hypertension and combined with diabetes are also assessed in accordance with corresponding international diagnostic guidelines.^25^, ^26^ Specifically, if the systolic blood pressure of the test population is greater than or equal to 140 mmHg, or the diastolic blood pressure is greater than or equal to 90 mmHg, or both, it is diagnosed as hypertension. The diagnostic standard for diabetes is mainly that the fasting blood glucose of test population is greater than or equal to 7.0 mmol/L, or the random blood glucose is greater than or equal to 11.1 mmol/L.

### 2.4 Study variables

Demographic characteristics includes age, gender, race, education, ratio of family income to poverty, smoking, drinking alcohol, body mass index (BMI), combined diseases (such as hypertension and diabetes), physical activity level and family history of osteoporosis. Laboratory examination includes the platelet count, neutrophil count, lymphocyte count, monocyte count, white blood cell count, red blood cell (RBC) count, hemoglobin (Hb), hematocrit (HCT), C‐reactive protein (CRP), creatinine (Cr), and 25‐hydroxyvitamin D [25(OH)D]. Based on stratification level standards reported in previous SII related literatures,^27^, ^28^ we have constructed the tertiles for sample analysis, and further divided it into three groups based on SII levels of less than or equal to 500, greater than 500 and less than or equal to 1000, and greater than 1000. Furthermore, the variables included in this present study are all deidentified and freely available to researchers via the webpage of https://www.cdc.gov/nchs/nhanes.

### 2.5 Statistical assessment

The differences of demographic characteristics and laboratory examination of the different groups are compared using the Student *t* test for continuous variables and Chi‐square test for categorical variables, as appropriate. Data are exhibited as mean ± *SD* for continuous variables and as frequency or percentage for categorical variables. The whole estimates are analyzed accounting for NHANES‐related sample weights. Then, we conduct multivariate weighted logistic regression analysis to assess the relationship between SII and osteoporosis, with 95% confidence intervals (CI) and odds ratio (OR) calculated. In logistic regression analysis, trend test (*P* for trend) is used to identify the linear trend relationship between the independent variable and the dependent variable. Considering the incidental influences of age, gender, race, weight, lifestyles, combined diseases and other factors, different adjustment groups are established and applied to identify the association within SII and osteoporosis. Receiver operating characteristics (ROC) curve is applied to screen the optimal cut‐off value of SII for indicating the occurrence of osteoporosis. In the process of analysis and calculation, the sensitivity ‐ (1‐specificity) can be used to obtain the Jorden index, and then the results can be sorted to obtain the maximum value of Jorden index, and corresponding result is the optimal cut‐off value. Next, we also divide the included people into two groups according to the SII below or equal to and higher than the optimal cut‐off value, and assess the OR and 95% CI of higher SII group in contrast to lower group. A *p* < .05 is considered statistically significant. R language 4.0.3 (R Foundation, Vienna, Austria) and SPSS 23.0 (SPSS Inc.) are used for overall data extraction and statistical assessment.

## 3 RESULTS

The demographic characteristics and laboratory examination of individuals based on the presence of osteoporosis is exhibited in Table 1. A total of 4625 middle aged and older individuals (2326 male and 2299 female) with a mean age of 62.55 ± 11.33 years are included in the final evaluation. We observe the individuals in osteoporosis group are older than those in nonosteoporosis group (*p* < .001), and osteoporosis group has higher ratio of female (*p* < .001) and non‐Hispanic White race (*p* < .001), lower ratio of family income to poverty (*p* < .001), BMI (*p* < .001), physical activity level (*p* = .012) than nonosteoporosis group. In addition, the osteoporosis group is also associated with higher SII (*p* = .024), lower RBC count (*p* < .001), Hb (*p* < .001), HCT (*p* < .001), and 25(OH)D (*p* < .001) than nonosteoporosis group.

**Table 1:** Demographic characteristics and laboratory examination of individuals based on the presence of osteoporosis.

Parameters	All (*n* = 4625)	Osteoporosis group (*n* = 435)	Non‐osteoporosis group (*n* = 4190)	*p* value
Age, years	62.55 ± 11.33	68.83 ± 10.12	61.90 ± 11.25	<.001
Gender, (*n*, %)				<.001
Male	2326 (50.3)	64 (14.7)	2262 (54.0)	
Female	2299 (49.7)	371 (85.3)	1928 (46.0)	
Race, *n* (%)				<.001
Mexican American	673 (14.6)	63 (14.5)	610 (14.6)	
Other Hispanic	297 (6.4)	24 (5.5)	273 (6.5)	
Non‐Hispanic White	2572 (55.6)	288 (66.2)	2284 (54.5)	
Non‐Hispanic Black	950 (20.5)	47 (10.8)	903 (21.6)	
Other race	133 (2.9)	13 (3.0)	120 (2.8)	
Education, *n* (%)				.005
Less than 9th grade	683 (14.8)	74 (17.0)	609 (14.5)	
9–11th grade	710 (15.3)	53 (12.2)	657 (15.7)	
High school grad or equivalent	1145 (24.8)	133 (30.6)	1012 (24.2)	
Some college	1147 (24.8)	102 (23.4)	1045 (24.9)	
College graduate or above	940 (20.3)	73 (16.8)	867 (20.7)	
Ratio of family income to poverty	2.76 ± 1.61	2.42 ± 1.51	2.79 ± 1.62	<.001
BMI, kg/m^2^	29.15 ± 6.36	28.09 ± 6.47	29.26 ± 6.34	<.001
Smoking (*n*, %)				.012
Yes	2464 (53.3)	207 (47.6)	2257 (53.9)	
No	2161 (46.7)	228 (52.4)	1933 (46.1)	
Drinking alcohol (*n*, %)				<.001
Yes	3125 (67.6)	232 (53.3)	2893 (69.0)	
No	1500 (32.4)	203 (46.7)	1297 (31.0)	
Combined with hypertension (*n*, %)				<.001
Yes	2265 (49.0)	249 (57.2)	2016 (48.1)	
No	2360 (51.0)	186 (42.8)	2174 (51.9)	
Combined with diabetes (*n*, %)				.595
Yes	808 (17.5)	80 (18.4)	728 (17.4)	
No	3817 (82.5)	355 (81.6)	3462 (82.6)	
Physical activity level (*n*, %)				.012
0 MET‐mins/week	1309 (28.3)	163 (37.5)	1146 (27.4)	
1–599 MET‐mins/week	794 (17.2)	97 (22.3)	697 (16.6)	
600–1199 MET‐mins/week	735 (15.9)	67 (15.4)	668 (16.0)	
≥1200 MET‐mins/week	1787 (38.6)	108 (24.8)	1679 (40.0)	
Family history of osteoporosis (*n*, %)				.719
Yes	1003 (21.7)	93 (21.4)	910 (21.7)	
No	3622 (78.3)	342 (78.6)	3280 (78.3)	
Platelet count, 10^3^ cells/µL	264.33 ± 70.46	265.97 ± 70.52	264.16 ± 70.46	.611
Neutrophil count, 10^3^ cells/µL	4.19 ± 1.60	4.25 ± 1.51	4.18 ± 1.61	.340
Lymphocyte count, 10^3^ cells/µL	2.14 ± 1.82	2.04 ± 1.01	2.15 ± 1.89	.225
SII	594.97 ± 372.25	633.37 ± 390.56	590.98 ± 370.11	.024
Monocyte count, 10^3^ cells/µL	0.56 ± 0.22	0.57 ± 0.19	0.56 ± 0.22	.420
WBC count, 10^3^ cells/µL	7.14 ± 2.70	7.10 ± 2.08	7.15 ± 2.76	.719
RBC count, million cells/µL	4.65 ± 0.50	4.44 ± 0.46	4.67 ± 0.50	<.001
Hb, g/dL	14.22 ± 1.53	13.67 ± 1.32	14.28 ± 1.54	<.001
HCT, %	41.73 ± 4.30	40.12 ± 3.82	41.90 ± 4.31	<.001
CRP, mg/dL	0.49 ± 0.89	0.54 ± 1.02	0.48 ± 0.87	.205
Cr, mg/dL	0.94 ± 0.41	1.03 ± 0.52	0.94 ± 0.39	.436
25(OH)D, nmol/L	73.91 ± 6.79	57.43 ± 3.94	76.12 ± 7.13	<.001

Abbreviations: 25(OH)D, 25‐hydroxyvitamin D; BMI, body mass index; Cr, creatinine; CRP, C‐reactive protein; Hb, hemoglobin, HCT, hematocrit; MET, metabolic equivalent; RBC, red blood cell count; SII, systemic immune‐inflammation index; WBC, white blood cell count.

Table 2 shows the samples and prevalence of osteoporosis in each tertile category, as well as the OR and 95% CI for osteoporosis according to the SII levels. Specifically, the prevalence of osteoporosis in the progressive tertile categories are 8.40%, 9.94%, and 11.97% (*P* for trend <0.001). Moreover, by taking lowest tertile category as a referent category, a higher level of SII is noticed to be positively linked to higher odd of osteoporosis. More importantly, this tendency is also not changed in the univariate model (*p* < .001), as well as the adjustments for parameters of age, gender, and physical activity level (Model 1, *p* < .001), parameters of diabetes, hypertension, and family history of osteoporosis (Model 2, *p* = .037), parameters of smoking, drinking alcohol, renal function, and 25(OH)D status (Model 3, *p* = .028), and all parameters (Model 4, *p* = .035).

**Table 2:** Odds ratios and 95% confidence intervals for the osteoporosis according to the systemic immune‐inflammation index levels.

SII	Osteoporosis, *n*	Prevalence, %	Univariate model	Model 1	Model 2	Model 3	Model 4
Categorical							
Tertile 1 (≤500)	188/2237	8.40	1 (Ref.)	1 (Ref.)	1 (Ref.)	1 (Ref.)	1 (Ref.)
Tertile 2 (>500, ≤1000)	190/1912	9.94	1.233 (0.900–1.689)	1.081 (0.761–1.513)	1.216 (0.887–1.667)	1.259 (0.915–1.723)	1.239 (0.903–1.712)
Tertile 3 (>1000)	57/476	11.97	1.483 (1.083–2.030)	1.313 (0.928–1.849)	1.455 (1.062–1.994)	1.509 (1.117–2.089)	1.485 (1.097–2.057)
*P* for trend		<0.001	<0.001	<0.001	0.037	0.028	0.035

*Note*: Model 1 adjust for the covariates of age, gender and physical activity level; Model 2 adjust for the covariates of diabetes, hypertension and family history of osteoporosis; Model 3 adjust for the covariates of smoking, drinking alcohol, renal function and 25(OH)D status; Model 4 is a fully‐adjusted model; SII, systemic immune‐inflammation index; 25(OH)D, 25‐hydroxyvitamin D.

**Figure 2:**
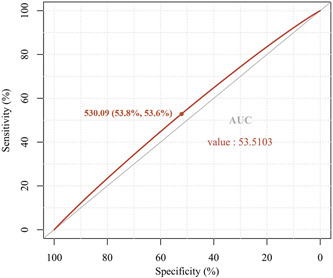
The SII of 530.09 (with 53.6% specificity and 53.8% sensitivity) is identified as an optimal cut‐off value for predicting the risk of osteoporosis through ROC curve, and the AUC is 0.535. AUC, area under the curve; ROC, receiver operating characteristic; SII, systemic immune‐inflammation index.

As exhibited in Figure 2, we also identify SII of 530.09 (with 53.6% specificity and 53.8% sensitivity) as an optimal cut‐off value for indicating occurrence of osteoporosis via ROC curve, and the area under curve is 0.535. Furthermore, as exhibited in Table 3, by dividing the included people into two groups in accordance with SII below or equal to and higher than optimal cut‐off value (530.09), logistic regression models indicate that people with SII ≥ 530.09 have 1.342‐fold increased odd of osteoporosis in the univariate model, 1.315‐fold increased odd in Model 1, 1.328‐fold increased odd in Model 2, 1.359‐fold increased odd in Model 3 and 1.302‐fold increased odd in Model 4.

**Table 3:** Odds ratios and 95% confidence intervals for the osteoporosis according to the optimal cutoff value of systemic immune‐inflammation index.

SII	Osteoporosis, *n*	Prevalence, %	Univariate model	Model 1^a^	Model 2^b^	Model 3^c^	Model 4^d^
Optimal cut‐off value^e^							
Below	201/2445	8.22	1 (Ref.)	1 (Ref.)	1 (Ref.)	1 (Ref.)	1 (Ref.)
Equal and above	234/2180	10.73	1.342 (1.101–1.636)	1.315 (1.073–1.628)	1.328 (1.086–1.641)	1.359 (1.117–1.648)	1.302 (1.053–1.592)

^a^ Model 1 adjust for the covariates of age, gender and physical activity level.

^b^ Model 2 adjust for the covariates of diabetes, hypertension and family history of osteoporosis.

^c^ Model 3 adjust for the covariates of smoking, drinking alcohol, renal function and 25(OH)D status.

^d^ Model 4 is a fully‐adjusted model.

^e^ The optimal cut‐off value of SII is 530.09; SII, systemic immune‐inflammation index; 25(OH)D, 25‐hydroxyvitamin D.

## 4 DISCUSSION

With the prolongation of the life span of population around world, osteoporosis and related osteoporotic fractures impose a growing burden on the social medical system.^1^, ^29^, ^30^, ^31^ Moreover, SII is an index derived from the number of various inflammatory cell subsets according to a specific equation.^32^ Since SII is less affected by various physiological conditions, it is considered to be more suitable as an inflammatory index to assess the inflammatory state of the body.^33^ However, the current studies on the relationship between SII and osteoporosis or BMD are still relatively sparse. Herein, the results of our NHANES‐based study suggest that among American population, the higher SII is positively related with the occurrence of osteoporosis among middle‐aged and older people. This result is consistent with the previous results of Tang et al.^28^ in predicting the risk of low BMD or osteoporosis among postmenopausal women using SII. However, it should be emphasized that in addition to targeting the postmenopausal women, our current study also includes the middle‐aged and older men with senile osteoporosis, and the predictive role of SII in the sum of these populations is also investigated. Hence, the novelty and advantage of this study lies in our further exploration of SII as a valuable and convenient inflammatory marker that could be used to predict the risk of osteoporosis among middle‐aged and older populations, whether it is the senile osteoporosis represented by males or the postmenopausal osteoporosis represented by females. Under the complex physiological state of aging and a variety of chronic diseases, inflammatory microenvironment among middle‐aged and older individuals is activated and the systemic immune function is relatively low.^34^ Moreover, there are a variety of inflammatory cells in bone marrow cavity. For example, dysfunctional lymphocytes can initiate the cascade reaction of inflammatory cytokines and chemokines, result in the aggregation of neutrophils and macrophages, and then disrupt the dynamic balance of bone, thereby inhibiting bone formation and induce bone resorption.^35^ Thus, there is a potential connection between the neutrophils, lymphocytes and platelets in blood and osteoporosis, and SII skillfully connects the three cells, which can more effectively reflect the overall level of immune and inflammatory state of whole body. In this present study, we notice that SII in osteoporosis group is significantly higher than that in nonosteoporosis group, indicating that there is a potential association between the high SII and high risk of osteoporosis, which is consistent with the results reported by Fang et al.^16^ and Tang et al.^28^ in the studies of correlation between SII and postmenopausal osteoporosis. In addition, by further establishing the logistic regression models and taking the lowest tertile category as a referent category, we notice that a higher SII is indicated to be positively linked to higher odd for osteoporosis (*P* for trend <0.001), which suggests that high SII is an important risk factor for the osteoporosis among middle‐aged and older people.

Furthermore, in view of pressing situation of global osteoporosis prevention, it is of great clinical importance to conduct the osteoporosis screening as early as possible among the middle‐aged and older people. As we all know, the current gold standard for the diagnosis of osteoporosis is DXA, but it has a certain lag.^36^ Compared with the DXA, SII can be easily and economically obtained from laboratory examination, and its reliability is also guaranteed.^16^ Based on this, we further identify the SII of 530.09 (with 53.6% specificity and 53.8% sensitivity) as an optimal cut‐off value for indicating the occurrence of osteoporosis. This value lies in that the SII of middle‐aged and older people should not exceed 530.09 to exhibit a potentially lower risk of osteoporosis. This is also one of the novelty and advantages of this study, which better assists in determining the risk of osteoporosis among middle‐aged and older people by determining the specific optimal cut‐off value. Thus, combined with high‐risk factors, such as the age and other comorbidities, clinicians could conveniently and quickly screen the individuals with osteoporosis among middle‐aged and older people through the SII and its optimal cut‐off value obtained in this study in the future clinical practice.

In addition, similar to the SII, the neutrophil‐lymphocyte ratio (NLR), monocyte‐to‐lymphocyte ratio (MLR) and platelet‐to‐lymphocyte ratio (PLR) are several other kinds of emerging indicators of chronic inflammation, which integrate the information from multiple leukocyte subtypes, have the advantage of good stability, and can reflect the response level of inflammation in body.^37^, ^38^, ^39^ A study on postmenopausal women conducted by Huang et al.^40^ revealed that NLR was an independent risk factor for the osteoporosis and can be used to predict the occurrence of osteoporosis. Moreover, in a cross‐sectional study of 316 patients with osteoporosis, Gao et al.^41^ found that MLR also had the early diagnostic value for osteoporosis, and was superior to NLR. Tang et al.^28^ also indicated that the postmenopausal women with a high level of SII or other inflammatory markers, such as NLR and product of platelet count and neutrophil count (PPN), should be aware of potential risk of osteoporosis. However, Lee et al.^42^ revealed that the predictive value of PLR for osteoporosis was relatively inapparent. Thus, we can find that the diagnostic value of different novel inflammatory indexes for osteoporosis is inconsistent in different studies. The latent reasons may be related to the different detection methods, relatively small sample size or differences in the race and diseases itself. Furthermore, the CRP can also be applied as both an inflammatory mediator and a nonspecific sensitive indicator of the immune inflammation.^43^ Ding et al.^44^ revealed that the increase of CRP in the elderly can result in the decline of BMD in the hip and spine, and the changes in CRP and other inflammatory factors (such as IL‐1β and IL‐6) are able to predict the bone loss and bone resorption. Thus, the targeted anti‐inflammatory therapy also has a certain potential in the prevention of osteoporosis.

Ultimately, although the novel findings as mentioned above, there are inevitably some drawbacks in this current study. First, the blood samples gathered by the NCHS are possibly not enough as the representative indexes for the long‐term laboratory examination of the population. Since the life span of blood cells is generally short, the continuous detection may be more abundant and reliable than the single detection. Second, studies based on NHANES are restricted by its self‐reporting property. Some items (collection of demographic data and the description of self and family information) may be relatively inaccurate, and there may be ineluctable recall and self‐reporting biases in the process of the overall data collection. Nevertheless, despite this, whole data collection process of NHANES is dependable and trusted, and has been verified by several previous researches.^45^, ^46^ Third, due to the different living habits and environments of individuals in different countries and ethnic groups, this study only includes the samples from the NHANES, and subsequent prospective investigations of more nationalities around world is also of great significance in the future.

## 5 CONCLUSIONS

This NHANES‐based research indicates that the higher SII is positively linked to the osteoporosis among middle‐aged and older people, and the SII should not exceed 530.09 for them to obtain a potentially lower risk of osteoporosis. In general, our study mainly focuses on the value of SII in timely diagnosis and prevention of osteoporosis in the middle‐aged and older people, although this evidence still needs to be verified in more nationalities around world in future.

## AUTHOR CONTRIBUTIONS

**Suli Zhang**: conceptualization; data curation; formal analysis; investigation; methodology. **Wenyan Ni**: conceptualization; data curation; formal analysis; investigation; methodology.

## CONFLICT OF INTEREST STATEMENT

The authors declare no conflicts of interests.

## ETHICS STATEMENT

This study was conducted in accordance with the principles of Declaration of Helsinki. The NHANES database used in this study did not contain identifiable nor protected the health information and was publicly available for download. In addition, this study was simply an exempt study and involved secondary processing data, which was approved by Research Ethics Review Board of NCHS and the written informed consent were also provided by the included participants.

## Data Availability

All data are available from the corresponding author upon request.
